# Radiographic Contrast-Media-Induced Acute Kidney Injury: Pathophysiology and Prophylactic Strategies

**DOI:** 10.5402/2013/496438

**Published:** 2013-09-16

**Authors:** Umar Sadat

**Affiliations:** Department of Surgery, Cambridge Vascular Unit, Addenbrooke's Hospital, Cambridge University Hospitals NHS Foundation Trust, Box 201, Cambridge CB2 0QQ, UK

## Abstract

Contrast-induced acute kidney injury (CI-AKI) is one of the most widely discussed and debated topics in cardiovascular medicine. With increasing number of contrast-media- (CM-) enhanced imaging studies being performed and growing octogenarian population with significant comorbidities, incidence of CI-AKI remains high. In this review, pathophysiology of CI-AKI, its relationship with different types of CM, role of serum and urinary biomarkers for diagnosing CI-AKI, and various prophylactic strategies used for nephroprotection against CI-AKI are discussed in detail.

## 1. Contrast-Induced Nephropathy

Contrast-induced acute kidney injury (CI-AKI) is one of the most widely discussed and debated topics in cardiovascular medicine. This is because an increasing number of individuals are exposed to iodinated contrast media (CM) during imaging-based investigations for either diagnostic or interventional purposes. The changing demographics of population especially increasing life expectancy has resulted in larger octogenarian population with comorbidities such as hypertension (HTN), diabetes mellitus (DM), and renal and cardiovascular disease, all of which predispose to renal impairment [1]. An increase in the incidence of CI-AKI is therefore not surprising. Thus, it is important that more attention is given in order to understand the aetiology of CI-AKI and devise novel diagnostic methods and formulate effective prophylactic and therapeutic regimens to reduce its incidence.

## 2. Problems of Definition of CI-AKI

Previously CI-AKI was defined as a condition characterized by acute and reversible renal failure of varying severity in patients exposed to intravascular CM and in the absence of other risk factors responsible for the change in renal function [2]. However, there were many problems with this definition. Firstly, renal failure may not be reversible [3]; secondly, there is no agreed threshold change in renal function to define a case; and thirdly, the CM may not be the sole but rather contributory factor to the renal impairment for a given patient. The problems with defining CI-AKI have hampered attempts to quantify its true burden and have led to conflicting estimates of its importance [4–6]. It would therefore be better to define a “case” in terms of clinical outcomes such as the need for dialysis or other intervention, rather than by the occurrence of a specific decline in the renal function.

Today, CI-AKI is widely defined as an absolute increase in serum creatinine (SCr) of 0.5 mg/dL (44 *μ*mol/L) or a relative increase of 25% from the baseline value, assessed 48–72 hours (hr) following (intravascular) administration of CM [7]. Even this definition has limitations as it fails to define CI-AKI in terms of clinical outcomes. Although such an increment in SCr concentration may not be clinically important, it does allow studies of reasonable sample size. If more sensitive definitions of CI-AKI (such as reducing absolute increase in SCr of 0.5 mg/dL to 0.3 mg/dL or a relative increase of 25% from the baseline value to 15%) were used, one can change a negative study to a positive one [6]. This would result in reduction of the required sample size for clinical trials but are these study end points truly valid? There are an abundance of studies with small patient populations in the CI-AKI literature which have demonstrated a reduction in the rise of SCr without a clear link to clinically meaningful outcomes. Larger studies are therefore required not smaller ones, statistically powered to assess differences in clinically relevant end points.

CM should be treated as potentially nephrotoxic agents that may become clinically important when combined with suitable comorbidity such as diabetic nephropathy [5] or with potentially nephrotoxic drugs such as nonsteroidal anti-inflammatory drugs (NSAIDs). This may render determination of CI-AKI burden more difficult but is likely to be productive in defining its pathogenesis and identify targets for specifically designed preventive measures [2]. The importance of the disease burden viewpoint is paramount for an individual patient who is about to receive CM. The patient will want to know the likelihood that he will suffer a clinically relevant decline in the renal function after contrast. An evidence based definition of CI-AKI in terms of clinical outcome is still awaited.

## 3. Relevance of Serum Creatinine Rise after CM Administration

SCr only rises out of normal range when greater than 50% of functioning renal mass is lost [8]. The clinical relevance of the increase in SCr from the baseline by 25% or 0.5 mg/dL (44 *μ*mol/L) has been questioned in the past. SCr has a curvilinear relationship with glomerular filtration rate (GFR). For patients at any level of renal function at baseline, SCr should double for GFR to reduce by 50% (i.e., 1.0-2.0; 2.0–4.0; 4.0–8.0 mg/dL) [9]. However, at each step the absolute change in GFR is progressively less. This is best illustrated by expressing the changes in terms of the reciprocal of the SCr (1/SCr). Here, the increases in the SCr represent stepwise decreases in the GFR of 50, 25, and 12.5%, respectively.

The opposite is however true for patients with chronic kidney disease (CKD). These patients have already raised SCr and low GFR. A small decrease in GFR results in large relevant changes in SCr. It can therefore be inferred from this that small changes in SCr in CKD patients exposed to CM are a result of clinically unimportant reduction in GFR [9]. However, this is not true as patients with CKD are at greater risk of developing CI-AKI than those with normal renal function or less severe renal impairment [10].

Patients who show seemingly modest changes are especially at higher risk of developing CI-AKI. In a retrospective analysis of >9700 patients without end stage renal disease (ESRD) undergoing coronary angiography [11], it was observed that for absolute SCr concentration, the strength of association between SCr increase and 30-day in-hospital mortality was more robust for small than for large SCr increase. The significantly increased odds for in-hospital mortality observed in this analysis indicate that a ≥25% rise in serum creatinine within 48 hr of a CM administration is indeed clinically important.

It should be remembered that like GFR, SCr is affected by age, gender, and changes in body mass. Hence a decline in GFR may be accompanied by proportional reduction in the body mass and SCr may therefore remain unchanged. Several formulas take into account age, gender, body weight, and race and include laboratory values of serum creatinine, blood urine nitrogen (BUN), and serum albumin. These are much more reliable and sensitive indicators of renal impairment than absolute laboratory values with their reference ranges; for example, Cockcroft-Gault equation estimates GFR by using SCr, body mass, gender, and age [12].

## 4. Incidence

The incidence of CI-AKI is variable. It is due to the variability in definitions of CI-AKI. This has also limited comparisons of the effectiveness of various prophylactic strategies across patient populations. These definitions have included relative increases of 25–100% or absolute increases of 0.25 to 1.0 mg/dL of SCr that occur within 48, 72, or 96 hr of CM administration [5, 13–16]. In other studies even more complex definitions have been used (such as an increase in SCr level of at least 25% from baseline, to at least 177 *μ*m/l (2 mg/dL) [17].

The prevalence of various risk factors in the research population, volume and type of CM used, and type of procedure all affect the incidence of CI-AKI. In patients without risk factors the incidence is ~2%. This may appear low but may amount up to 60 million cases/year in developed countries, due to high volume of CM-enhanced radiological examinations [18]. In patients with mild-to-moderate renal impairment and DM, incidence of CI-AKI may range between 9 and >50% [5, 19].

A prospective study of 2262 patients, aimed at determining the contribution of iatrogenic factors to the development of hospital acquired renal insufficiency, reported that some degree of renal impairment developed in 4.9% cases [20]. Exposure to CM was the third most frequent cause of renal impairment (12%), preceded by renal hypoperfusion and major surgery. Another study of 4622 patients reported a 7.0% prevalence of hospital acquired renal insufficiency [21], with 29% of these cases having CI-AKI preceded by renal hypoperfusion and use of nephrotoxic drugs.

Despite advances in our understanding of CI-AKI, development of improved CM, and prophylactic strategies, it is quite intriguing that the incidence of CI-AKI has not changed during this time period [22].

## 5. Pathophysiology of CI-AKI

**Figure 1:**
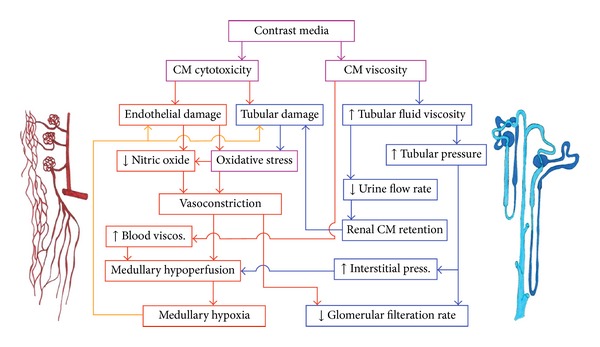
Major mechanisms of CI-AKI: CM effects that primarily affect the nephron are depicted in blue (see stylized nephron with glomeruli, tubules, and collecting duct at the far right), effects that primarily affect blood perfusion and tissue oxygenation are depicted in red (see stylized vasculature including afferent and efferent arterioles, tufts of glomerular capillaries, peritubular capillaries, and descending vasa recta (DVR) at the far left), and CM properties and effects that affect both are in pink. The orange arrows indicate a feedback that may result in a vicious cycle: medullary hypoxia aggravates cellular damage that, by several factors, increases vasoconstriction (reproduced with permission [23]).

To date, our understanding of the underlying pathophysiology remains incomplete [23]. It is believed to include direct cytotoxic effects of CM on renal tissue, altered renal hemo- and tubulo-dynamics, and the interaction between them (Figure 1). 

### 5.1. Cytotoxic Effects of CM-Cell Culture Models

CM are discussed in detail later, but it is important to mention that all CM (regardless of their ionicity and osmolality [24]) are cytotoxic due to toxicity of iodine [25], at similar concentrations of iodine [26].

Iodine has well known cytotoxicity to bacteria. Free, noncomplexed iodine (I_2_) and its polarized form (H_2_O  I^+^) are considered responsible for its microbicidal action [27]. The proposed mechanism includes interaction of iodine with amino acids found in cell membrane proteins, damaging them and causing loss of cell membrane integrity. Through similar processes, it may exert its direct toxicity on renal vascular and tubular cells.

Toxicity of iodinated CM in various cell culture and in vivo animal models has been reported such as renal epithelial (tubular) cells [24, 28], renal endothelial cells [28], and mesangial cells [29, 30]. Signs of cell damage were evident in all these studies, including damage of tubular tight junctions [31]. Other studies have shown that CM impair cellular function such as reduced nitric oxide production [32] and increased endothelin production [33], both promoting vasoconstriction. Aggravation of this damage has been observed in the presence of glucose [34]. It does so independently of its osmotic effect, by worsening the oxidative stress. This may explain higher risk of CI-AKI in patients with DM.

### 5.2. Effect of CM on Renal Vascular Function

Various studies have shown different vascular responses to CM exposure: from constriction to dilatation to biphasic response (dilatation followed by constriction) depending on the vessel and species being studied [35]. However, hypoperfusion of the medulla and hypoxia has been particularly observed in models of CI-AKI [36–38]. The outer medulla is especially vulnerable to hypoxia. This is due to high absorptive activity in medulla which increases oxygen requirements. Oxygen delivery to the outer medulla is low due to (1) arteriovenous shunt diffusion and (2) great distance between DVR which bring blood to the medulla. DVR are thin vessels (12–15 *μ*m in diameter) which originate from efferent arterioles of juxtamedullary nephrons [39]. Endothelium of DVR is surrounded by pericytes which are capable of active contraction (especially in response to angiotensin II, endothelin, and reduced nitric oxide [NO]) and thus of controlling medullary blood flow [39]. Intraluminal CM application causes constriction of DVR by reducing NO bioavailability, which is further increased in response to angiotensin II [40]. Cytotoxic effect of CM on endothelium may also increase the oxidative stress or be partly caused by it [41], causing constriction of DVR [40]. These effects of CM are independent of the ionicity, osmolality, or viscosity of CM [42]. Given the size of DVR, their constriction can physically block the flow of blood cells. Diminished plasticity of red blood cells due to the effect of CM on their morphology, aggregation, and deformability can also have a negative rheological effect [43–48].

Besides medullar vasoconstriction, CM may also cause vasoconstriction in cortex by shifting the balance between vasodilatory and vasoconstrictor factors towards vasoconstriction [23, 35]. The degree of cortical vasoconstriction may differ significantly from the medulla [49]. Cortical vasoconstriction, more precisely preglomerular constriction, is a major cause of CM-induced reduction in GFR. Preglomerular constriction may also reduce medullary blood flow as DVR emerge from efferent arterioles.

### 5.3. Effect of CM on Renal Tubules

Increased viscosity of CM can increase the tubular fluid viscosity [50], impeding its flow leading to prolonged renal retention of CM [49]. CM with same osmolality as plasma have higher viscosity compared to low osmolality CM and have been observed to increase the tubular fluid viscosity [51]. In addition, CM can cause loss of tubular cell membrane and cell damage by above-mentioned mechanisms.

## 6. Choice of CM

CM are classified as ionic or nonionic. Ionic media dissociate in water. Nonionic media do not dissociate in water but yet have the desirable property of being hydrophilic (water soluble). The ratio of iodine atoms to dissolved particles is an important characteristic of CM. A higher ratio is desirable, since more iodine means better opacification and fewer dissolved particles mean a lower osmotic effect. Based on this ratio, various CM are classified as follows.

*(1) High Osmolality Contrast Media (HOCM): First Generation.* Their ratio is 1.5 (i.e., for every 3 iodine atoms two particles are present in solution (ratio 3 : 2). Their osmolality ranges from 1500 to 2000 mOsm/kg, whereas that of human plasma is 290 mOsm/kg. These constitute the first generation of CM. Being ionic monomers (e.g., Diatrizoic acid) they had an ionic carboxyl group attached to the first carbon of the iodine-containing benzene ring.

*(2) Low Osmolality Contrast Media (LOCM): Second Generation.* Their ratio is 3. Their osmolality ranges from 600 to 1000 mOsm/kg (i.e., 2-3 times that of human plasma), at an iodine concentration of 300 mg/mL. These constitute second generation of CM. They can be nonionic monomers lacking carboxyl group; being nonionic for every three iodine atoms, only one is present in the solution (ratio 3 : 1). They may be ionic dimmers, which have a slightly lower osmolality in solution than the nonionic monomers. They dissociate in solution; for 6 iodine atoms there are two particles in solution (ratio 6 : 2). LOCM currently available for clinical use are the ionic dimer ioxaglate (Hexabrix), and the nonionic monomers, iohexol (Omnipaque), iopamidol (Niopam), Iomeprol (Iomeron), iopromide (Ultravist), ioversol (Optiray), iobitridol (Xenetix), and iopentol (Imagopaque).

*(3) Isoosmolar Contrast Media (IOCM): Third Generation.* The ratio is 6 (for 6 iodine atoms, one is in solution). Being isoosmolar they have the same osmolality as plasma (280–290 mOsm/kg). These are dimers, with two molecules of CM linked together by a shared side chain, giving them a higher viscosity than the previous generation CM. The only IOCM approved for intravascular use are iodixanol (Visipaque), which is isoosmolar with blood at an iodine concentration of 320 mg/mL.

## 7. Current Use of Iodinated CM

HOCM have been completely replaced by LOCM in western countries due to lower incidence of side effects from LOCM with no difference in image quality. The incidence of mild and moderate contrast reactions is higher for HOCM (6%–8%) than for LOCM (0.2%), but the incidence of severe reactions remains similar [52]. These include anaphylactoid reactions and cardiovascular decompensation more common while using HOCM [53]. In patients with normal renal function, HOCM have been found to be safe and associated with little decline in renal function. However, in patients with renal insufficiency (estimated GFR [eGFR] <60 mL/min), HOCM are associated with nearly twofold higher incidence of CI-AKI [54]. This analysis was performed on studies that did not routinely include prophylactic volume expansion or other pharmacological prophylaxis.

Whether IOCM are less nephrotoxic in comparison to LOCM remains contentious [22]. Sharma and Kini reported data from 560 patients with chronic renal impairment, 245 receiving iopamidol (LOCM), 209 receiving iodixanol (IOCM), and 106 receiving iohexol (LOCM). Iohexol use had highest incidence of CI-AKI (25%) followed by iopamidol (13.5%) and iodixanol (11%). There was significant difference in the incidence of CI-AKI between iohexol and iodixanol (*P* = 0.001) and between the two LOCM (*P* = 0.02), whereas difference between iodixanol and iopamidol was not significant (*P* = 0.27).

Solomon published a systematic review of seventeen RCTs on 1365 patients with renal impairment receiving intra-arterial iodixanol, iopamidol, or iohexol [55]. The risk of CI-AKI was similar with the iodixanol and iopamidol but significantly lower compared to iohexol. The incidence of CI-AKI with iohexol was also significantly higher than with iopamidol, despite having similar osmolalities. These data suggest that factors other than osmolality play a significant role in the pathogenesis of CI-AKI, at least for agents with osmolalities of 800 mOsm/kg or less. Later the same author performed a metaregression analysis of 22 RCTs, reporting that highest incidence of CI-AKI occurs in patients receiving iohexol and the lowest incidence in patients receiving iopamidol, even when corrected for other CI-AKI risk factors [56].

Heinrich et al. reported a meta-analysis of 25 trials using strict inclusion criteria by defining CI-AKI and their overall quality score [57]. Iodixanol was not associated with a significantly reduced risk of CI-AKI compared with the LOCM pooled together. However, in patients with intra-arterial administration and renal insufficiency, iodixanol was associated with a reduced risk of CI-AKI compared with iohexol, whereas no significant difference between iodixanol and other LOCM could be found. An interesting response to this meta-analysis was made by Capasso, most importantly highlighting that all past trials enrolled small numbers of patients, and most lacked sufficient power to determine potentially subtle differences between CM [58]. To illustrate this point, in a hypothetical trial comparing two CM that assumes a baseline rate of CI-AKI of 10%, a 20% effect size, *α* of 0.05, and 90% power, more than 4000 patients would be required in each arm. Viewed slightly differently, a clinical trial that enrolled the same number of patients with data available on the development of CI-AKI (*n* = 2654) as were included in this meta-analysis would have less than 50% power with these same statistical and clinical assumptions. They concluded that large adequately powered trials that use serious patient-centered outcomes are needed in the highest risk patients to address this important question. Capasso and Weisbord responded with the explanation that clinically relevant outcomes (e.g., death or dialysis) induced by CM are extremely rare after intravenous application [58]. Thus, thousands of patients would have to be included to demonstrate a difference between CM after intravenous application, and if such study were performed, the true clinical importance of this difference would have to be questioned. Since their meta-analysis indicates that there are no heterogeneity and no evidence for a clinically relevant difference after intravenous use, at present guidelines on the use of CM have to be established on the basis of the evidence currently available. In a recent meta-analysis by Reed et al. similar results have been reported [59].

Considering all the current available data, CM safety committee (CMSC) guidelines recommend the use of LOCM and IOCM in patients with risk factors for CI-AKI [60, 61]. There is consensus that all CM, including the isoosmolar dimer iodixanol, are potentially nephrotoxic in patients with risk factors. Provided HOCM are avoided, relying just on using certain CM to reduce the risk of CI-AKI can be misleading and may give a false sense of security. The safest approach to minimize the risk of CI-AKI remains the use of the lowest possible dose of either LOCM or IOCM and offering the patient an adequate hydration regime.

## 8. Route of CM Administration

There are no head-to-head trials comparing intravenous and intra-arterial use of CM. Intra-arterial administration compared to intravenous use has been believed to have higher incidence of CI-AKI, particularly when used above the level of renal arteries due to more contrast load delivered to kidneys. A 5% incidence of CI-AKI has been reported by Katzberg and Newhouse, with intravenous use of CM in patients undergoing contrast-enhanced CT [62]. The clinical studies reviewed by the authors did not include many patients with marked renal impairment (CKD 4 and 5). They further reviewed 1,075 patients with renal impairment in prospective CT trials and found that none required dialysis and there was no mortality [63]. In contrast, with intra-arterial CM administration the need for dialysis ranged from 0.7% in general population [64] to 7% in CKD patients [65].

The CM safety committee consensus is that the risk of CI-AKI is significantly lower following intravenous CM administration compared to intra-arterial [66]. They also concluded that an eGFR <60 mL/min/1.73 m^2^ is considered a risk factor for intra-arterial CM administration compared to eGFR <45 mL/min for patients with CKD stages 3, 4, and 5 undergoing CM-enhanced CT [66].

Following these consensus guidelines, a head-to-head comparison between intravenous and intra-arterial iodinated CM administration was reported in patients undergoing angiography of aortofemoral arteries [67]. Patients (*n* = 264) underwent contrast-enhanced CT imaging 3–14 days before angiography, thereby exposing them to two loads of CM. If intra-arterial CM use had stronger association with CI-AKI, this would result in this population. However, there was no difference between incidence of CI-AKI between the two groups (7.6% after intravenous iodixanol administration and 8.7% for angiography with intra-arterial iodixanol or LOCM (*P* = 0.64)). In the 143 patients who received only iodixanol for both procedures, incidences of CI-AKI were comparable after intravenous (7.0%) and intra-arterial (5.6%) administration (*P* = 0.62). They concluded that intravenous route may be as nephrotoxic as intra-arterial route. They also suggested that most intra-arterial injections (besides coronary and subclavian arteries) are mainly intravenous for the kidney, that is, have to pass through venous system before reaching kidneys (e.g., in carotid, celiac mesenteric, distal aortic, and iliofemoral arterial imaging). Another exception to this is left ventriculography. However, even then only a minor portion will reach the kidneys directly during the first pass through the aorta, that is, about 20% of cardiac output or 6–8 mL of an injected volume of 30–40 mL. This corresponds to only 2-3 gm of iodine (anticipated concentration 350 mg iodine/mL) of a total mean dose commonly ranging between 40 and 90 gm iodine during coronary procedures [68]. Direct CM exposure to kidneys is highest in suprarenal, juxtarenal, or selective renal artery injections. They also stated that because CT studies use lesser quantity of CM (commonly 25–50 gm of iodine) [69] compared to 40–90 gm of iodine in intra-arterial injections, the reported incidence of CI-AKI is therefore lower. In similar doses, incidence may be within similar range, reported by two CT studies (27.8% [70] and 42% [71] in patients with raised baseline SCr given iopromide and by angiography studies using a mean CM dose of 50 gm of iodine (The Iohexol Cooperative Study 12–33% for iohexol and 27.0–47.7% for diatrizoate [72]; Nephric Study 26% (for iohexol) [14]). A recent meta-analysis of 11 RCTs including 2,210 patients with intra-arterial route and 7 RCTs including 919 patients with intravenous route has reported that intra-arterial use of iodixanol (IOCM) significantly decreased the risk of CI-AKI (*P* = 0.01, heterogeneity *P* = 0.14), when compared with a pool of LOCM [73]. There was however no significant benefit with intravenous application (*P* = 0.27, heterogeneity *P* = 0.40).

## 9. Dose of CM

There had been mixed reports as to whether or not volume of CM administered is a determinant of CI-AKI. Smaller prospective studies showed lack of association [74] whereas retrospective analyses of large databases confirmed this association [75]. This conflict arose because prospective studies were underpowered to establish this relationship. A contrast volume limit of 5 mL/kg divided by SCr (to a maximum of 300 mL) has been proposed as a way to predict CI-AKI in patients receiving CM [76]. This was validated in 115 patients with CKD (SCr > 1.8 mg/dL) undergoing angiography. Patients who received >5 mL/kg/SCr had a higher incidence of CI-AKI. This formula was applied retrospectively in 16,592 patients undergoing cardiac catheterization to determine its utility in predicting the risk of postprocedural dialysis. Patients who received a volume of contrast in excess of 5 mL/kg/SCr had 6-fold more likelihood to develop nephropathy requiring dialysis [75].

Another development has been the use of volume-to-creatinine clearance ratio (v/CrCl) [77], as an index for prediction of an abnormal increase in postpercutaneous coronary intervention creatinine [68], rather than considering of contrast volume or underlying renal function alone. A ratio of the CM volume to the creatinine clearance below 3.7 has been suggested as a safe limit [68].

Nyman et al. have proposed CM dose (gm iodine to eGFR ratio) as an index to assess CI-AKI risk (at a gm iodine/eGFR ratio <1 the risk of CI-AKI was 3%, while it was 25% at a gm iodine/eGFR ratio ≥1) [78, 79].

These recommendations cannot be directly applied to intravenous use such as in enhanced CT, but they give a pointer for future studies. A “safe” dose does not exist and even very limited doses (less than 100 mLs) of CM may cause CI-AKI in high-risk patients [80]. There is general consensus now that in all patients, only the minimum amount of CM necessary to answer the clinical question should be used [66].

## 10. Biomarkers of Acute Kidney Injury

SCr remains the most commonly used renal biomarker for defining CI-AKI in majority of clinical trials. Its efficacy and limitations have been discussed earlier. Here we discuss briefly other commonly used renal biomarkers for assessing acute kidney injury. 

### 10.1. Retinol Binding Protein (RBP)

Retinol binding protein is a 21 kDa glycoprotein which is synthesized in the liver. It is responsible for transporting vitamin A (as retinol) from liver to the other tissues. Vitamin A mobilization from liver and its delivery to peripheral sites of action are a highly regulated process. It is particularly controlled by processes that regulate the rates of production and secretion of RBP by liver [81]. Human RBP has one binding site for one molecule of retinol. RBP also interacts with another protein, plasma prealbumin, and normally circulates as 1 : 1 molar RBP-prealbumin complex [82]. The biological half-life of RBP is short (4 hr in normal subjects) but increased by 10–15-fold in patients with severely impaired renal function [83]. The major part of its turnover can probably be accounted for by glomerular filtration and subsequent tubular metabolism [83]. RBP is reabsorbed by the proximal tubules where it is catabolised. Normally very small quantities of RBP are found in the urine. In tubular damage more than 100 mg/day may be excreted in the urine. RBP loss is greater in nephropathies with tubular lesions than glomerular lesions because of a failure of reabsorption and metabolism by proximal tubular epithelial cells.

Urinary excretion of RBP has been suggested to be a sensitive index for use in screening for tubular proteinuria [84–86]. Bernard et al. monitored patients with renal tubular damage secondary to multiple injuries, rhabdomyolysis, antibiotic treatment, or poisoning by various chemicals such as solvents, heavy metals, or pesticides. In almost all cases, RBP proved to be a more sensitive index of renal tubular damage [87]. In addition, Roberts et al. observed RBP to be a sensitive indicator of acute renal insufficiency in infants after birth asphyxia—a setting where interpretation of SCr is particularly problematic as it reflects maternal serum concentration to a significant extent [88]. Recently it has been used to assess renal injury due to ischemia reperfusion in patients undergoing aortic aneurysm repair [89, 90] and also for assessment of CI-AKI [91]. Serum RBP levels are depressed in vitamin A deficiency and urinary levels may yield a false negative in this setting [92].

### 10.2. Albumin Creatinine Ratio (ACR)

Albuminuria is a direct consequence of renal glomerular/tubular injury and increases with glomerular dysfunction [93, 94]. It is a known marker for progression of CKD and also a risk factor for cardiovascular disease [95]. Spot urine albumin: creatinine ratios are a reasonable surrogate for 24 hr urine albumin excretion rates [96]. According to the National Institute for Health and Clinical Excellence (NICE) guidelines ACR measurement is the recommended first line test for proteinuria detection [97]. This is because ACR offers greater sensitivity for the detection of lower, but clinically significant levels of proteinuria [97]. As CM can cause renal vascular and tubular damage, resulting proteinuria may indicate CI-AKI. Using this hypothesis, Levin et al. showed that CM would result in worsening of ACR and NAC, being a potential nephroprotective agent with antioxidant properties, and would result in its improvement [98]. Worsening of ACR due to CI-AKI in patients undergoing endovascular aortic aneurysm repair has also been reported [91].

### 10.3. Cystatin C (Cys C)

It is a 13 kDa nonglycosylated protein belonging to the Cystatin superfamily of cysteine endopeptidase inhibitors [99]. Being a proteinase inhibitor it is involved in the intracellular catabolism of peptides and proteins. It is also a very important extracellular inhibitor of cysteine proteases. It is produced by all nucleated cells and released into the blood stream at a constant rate [100]. Lack of significant protein binding and small molecular size favors free glomerular filtration (>99%). It is reabsorbed by proximal renal tubules where it is catabolized. It is not secreted by renal tubules. These features make serum Cys C a favorable biomarker for assessing early glomerular dysfunction, rather than tubular. In a meta-analysis, it was reported to be superior to SCr as a marker of glomerular filtration [101]. This was attributed to its ability to be not significantly affected by nonrenal factors such as age or body mass. Soon after, a large cross sectional study of 8000 patients revealed that numerous nonrenal factors such as older age, male gender, greater weight, greater height, current cigarette smoking, and higher serum C-reactive protein can be associated with elevated serum Cys C levels [102].

As previously mentioned, Cys C is completely reabsorbed by proximal tubules and therefore the physiological urinary Cys C concentrations are extremely low. Following renal tubular injury, the absorption of Cys C is impaired leading to elevated urinary Cys C levels [103]. Proteinuria however increases the urinary secretion of Cys C [104]. In a recent meta-analysis, it has been reported that urinary Cys C excretion has only moderate diagnostic value, whereas serum Cys C appears to be a good biomarker in the prediction of acute kidney injury [105].

### 10.4. Neutrophil Gelatinase-Associated Lipocalin (NGAL)

Human NGAL is a 25 kDa protein covalently bound to gelatinase from human neutrophils [106]. It is also expressed at very low concentrations in several human tissues such as kidney, lungs, trachea, stomach, and colon [107]. Injury to the epithelium and inflammation increase expression of NGAL such as in patients with chronic obstructive pulmonary disease [108], cystic fibrosis [109], and rheumatoid arthritis [110]. NGAL levels increase in viral and bacterial infections [111]. Upregulation of NGAL following renal tubular damage secondary to renal injury has also been reported [112–117].

Plasma NGAL is freely filtered by glomerulus and is largely absorbed by the proximal tubules [118, 119]. Urinary excretion of NGAL occurs when its reabsorption is impeded by renal tubular injury and/or concomitant increased NGAL synthesis. Increased NGAL synthesis in distal nephron has also been reported [120], suggesting that elevated urinary NGAL can result from both proximal and distal tubules. In a meta-analysis, NGAL has been reported to have diagnostic value for acute kidney injury with serum and urinary NGAL having similar diagnostic accuracy [121]. In the absence of diagnostic increases in SCr, NGAL can also detect patients with subclinical acute kidney injury who have an increased risk of adverse outcomes [122]. In contrast to SCr which increases when more than 50% of renal function is lost [8] and also influenced by hemodynamic prerenal causes of acute kidney injury, NGAL has been reported to represent intrinsic renal damage (at the level of nephron) confirmed by renal biopsy [123, 124]. Increased levels of urinary NGAL have been reported as early as 1–3 hr after renal insult [116, 125, 126] with peak concentration in urine and serum reached at 6 hr [127].

Three isoforms of NGAL have been isolated [128]: 25 kDa monomer, 45 kDa homodimer, and 135 kDa heterodimer. For neutrophils, homodimer isoform is specific [128] but to some extent the monomeric isoform is also expressed [129]. Monomer and to some extent heterodimer isoforms are synthesized by renal tubule epithelial cells [130, 131]. A bioassay which would differentiate these isoforms would therefore be most useful to specify the site of NGAL generation, as nonrenal sources of NGAL may act as confounders while interpreting NGAL with regard to acute kidney injury. Very recently, an immunoassay has been developed to aid identification of different NGAL isoforms [66]. Other factors which may influence NGAL concentration and hence interpretation of results include age, gender, markers of inflammation [132, 133], and liver function [134].

## 11. Prediction Models for Assessing Risk of CI-AKI

There is a clinical need to predict the probability of development of CI-AKI, in order to support decisions about formulating optimum prophylactic and therapeutic regimens. Risk factors that can be identified following above discussion include CKD, DM, use of nephrotoxic agents including CM and medications such as NSAIDs, factors which reduce renal perfusion such as preprocedural hemodynamic instability, and volume depletion. The effect of these risk factors is additive and risk of CI-AKI increases with an increase in the number of risk factors. This was reported initially by Cochran et al. 3 decades ago, proposing a clinical risk model that an increase in the number of concomitant risk factors increases the change in SCr levels [135]. Other investigators have also consistently reported the relationship between different risk factors and an increased risk of CI-AKI in peripheral arteriography [136–138] and coronary angiography [64, 139, 140]. The additive nature of risk has allowed the development of prognostic scores to facilitate risks prediction of CI-AKI in clinical practice. A risk model combines ≥2 risk factors (patient-specific and/or radiological procedure related) to enable reliable prediction of clinical outcomes such as likelihood of developing CI-AKI, change in SCr levels from baseline, or need for renal replacement therapy. To be useful, a risk prediction model should have predictive ability in populations other than one used for development. This validation process of a model is important for it to be labeled robust. Various CI-AKI risk prediction models have been proposed [64, 75, 139–146]. All these models have been developed from large databases of patients undergoing coronary angiography, with data usually divided into derivation set and validation set. Due to the retrospective nature of validation with no independent external validation in a multicentre setting using a large prospective patient registry, the robustness and hence adoption of these risk prediction models for clinical practice are yet to be established.

## 12. Prevention of CI-AKI

### 12.1. Volume Expansion

#### 12.1.1. Intravenous Hydration

Adequate hydration of patients undergoing CM-enhanced imaging studies was suggested approximately 40 yrs ago [147], based on propositions of previous studies [148, 149]. This was due to observation that dehydration would exacerbate renal insufficiency in a patient exposed to CM [150, 151]. The beneficial effects of hydration were initially reported in early 1980s by studies comparing outcomes of hydrated patients with historical controls [152–154]. These reports were supported by the first RCT in 1994, concluding that patients with chronic renal impairment benefit more from intravenous (0.45%) saline administration (for 12 hr before and 12 hr after angiography) compared to saline plus mannitol or furosemide [155]. Since then, various RCTs have confirmed the benefit of intravenous normal saline (0.9%) hydration, started 12 hr before to 12 hr after CM injection [16, 156, 157] in preventing CI-AKI over 0.45% saline [16] or a fluid bolus (300 mL) during CM administration only [158]. The rate of infusion has been reported as 1 mL/kg/hr [17, 159]. This regimen, however, is impractical in outpatient setting.

CM safety committee recommends an intravenous regime of 1.0-1.5 mL/kg/hr for at least 6 hr before and after CM administration [66].

#### 12.1.2. Oral Hydration

In an effort to overcome the limitations of outpatient intravenous hydration, investigators have assessed the use of preprocedure oral hydration followed by postprocedure intravenous hydration in patients admitted for catheterization studies on the day of procedure. In an RCT on patients with mild-to-moderate renal impairment, Taylor et al. reported an effective protocol comprising of preangiography oral hydration (1,000 mL clear fluids over 10 hr) followed by 6 hr of intravenous hydration (0.45% normal saline solution at 300 mL/hr) beginning just before CM exposure [160]. The results were as good as with overnight intravenous hydration (0.45% normal saline solution at 75 mL/hr for both 12 hr before and after angiography). A limitation of this protocol can be high infusion rate (300 mL/hr) after procedure for patients with left ventricular impairment. Trivedi et al. reported somewhat different experience when they observed that patients with unrestricted oral hydration had more chances of acute renal insufficiency than those receiving normal saline for 24 hr (at a rate of 1 mL/kg/hr) beginning 12 hr prior to scheduled catheterization (*P* = 0.005) [156]. In this study, however, there was no set protocol for oral hydration for patients to follow, which perhaps could have contributed to its ineffectiveness.

Later, Dussol et al. randomized 312 patients with CKD to receive either per oral sodium chloride (NaCl) (dose: 1 gm/10 kg bodyweight/day for 2 days before the procedure), intravenous normal saline 15 mL/kg for the 6 hr before the procedure (control arm), theophylline, or furosemide in addition to the treatment given to patients in the control arm [161]. Oral saline hydration was found as effective as intravenous saline hydration in preventing CI-AKI. Very recently, Wróbel et al. have reported that oral hydration (commercially available still mineral water or boiled water) administered at 1 mL/kg/hr between 6 and 12 hr before the procedure and continued up to 12 hr after procedure, and intravenous hydration with normal saline has similar effects on renal function in high-risk patients undergoing coronary angiography and angioplasty [162]. The fluid volume was reduced by 50% in patients with heart failure.

#### 12.1.3. Sodium Bicarbonate-Based Hydration

Acidic environment which is typical of tubular urine promotes free radical production [163] while high pH of normal extracellular fluid inhibits it [164, 165]. Since CM administration increases the oxidative stress and increases generation of free radicals and reactive oxygen species (ROS), alkalinizing renal tubular fluid with bicarbonate seems a logical strategy to reduce renal injury [166]. This potentially beneficial effect of sodium bicarbonate is not surprising in light of pH conditions within the nephron [167]. As a consequence of active reabsorption the tubular bicarbonate concentration decreases (to about 6 mEq/L) and the tubular fluid pH is ~6.5 near the end of the proximal tubule in the medulla [168]. In the descending loop of Henle, water and chloride are passively reabsorbed. This increases urine pH to ~7.4 at the tip of the papilla, but this region is spared from contrast nephropathy [169], suggesting that higher pH is protective. Also important is the observation that outer medulla is most susceptible to CI-AKI [159] and has acid pH [164] which favors activity of ROS. Superoxide, an ROS generated by ischemia, might react with medullary NO to form the potent oxidant peroxynitrite [158]. At physiologic concentrations, bicarbonate scavenges peroxynitrite and other ROSs generated from NO [170]. Thus, several oxidant mechanisms of renal injury might be disrupted by sodium bicarbonate.

The beneficial effect of higher proximal tubular pH is supported by a report that acetazolamide, a carbonic-anhydrase inhibitor which blocks proximal tubular bicarbonate reabsorption, is protective in contrast-induced renal failure [171]. Assadi suggested that the increase in urine pH to greater than 7.0 was the sign of excretion of a substantial amount of bicarbonate and, consequently, the efficiency of alkalinization. However, in that study, urine pH greater than 7.0 was achieved in only the group receiving acetazolamide, and postbolus urine pH in the bicarbonate group was 6.4 ± 0.5. In a meta-analysis of studies assessing effectiveness of sodium bicarbonate, it was observed that 6 studies monitored the degree of alkalinization (pH of urine or blood) [167, 172–176]. All but one [174] found a significant increase in pH, which in fact was the only one among them not to find a benefit of sodium bicarbonate. Therefore, it could be hypothesized that sodium bicarbonate should be dosed to achieve urinary alkalinization.

Merten et al. reported first study on the use of sodium bicarbonate in humans as a nephroprotective agent [167]. Patients received 154 mEq/l of either NaCl (in 5% dextrose H_2_O) or sodium bicarbonate (in dextrose H_2_O), as a bolus of 3 mL/kg/hr for 1 hr before iopamidol contrast, followed by an infusion of 1 mL/kg/hr for 6 hr after the procedure. CI-AKI occurred in 8 patients (13.6%) infused with NaCl but in only 1 (1.7%) of those receiving sodium bicarbonate (*P* = 0.02). Since then many RCTs have compared efficacy of sodium bicarbonate with saline hydration in prophylaxis against CI-AKI. These have been reviewed in multiple meta-analysis [177–183], which have concluded that sodium bicarbonate-based saline hydration is superior to saline hydration only. Heterogeneity and bias have been a limitation of such pooled analyses.

The most common protocol used in above studies is 3 mL/kg/hr for 1 hr before and 1 mg/kg/hr for 6 hr after procedure [61], although the dose of bicarbonate should be increased until urine pH becomes alkaline. This protocol is quicker than intravenous isotonic hydration protocol and hence more practicable in outpatient setting. This is also the recommendation of CM safety committee.

In 2009, Tamura et al. reported that a single-bolus intravenous administration of sodium bicarbonate (20 mEq) 5 minutes before CM exposure along with standard hydration with NaCl (for 12 hr before procedure to 12 hr after procedure) is more effective against CI-AKI than standard hydration alone in patients with mild renal insufficiency [184]. Urinary and blood alkalinization was found to be significant in patients receiving sodium bicarbonate. Following this study, Meier and Gurm performed a stratified meta-analysis [185] based on their previously published analysis [183]. Studies with bicarbonate infusion >1 hr before contrast application were defined as “long-infusion” and those with ≤1 hr infusions as “brief infusion” protocols. Control group received normal saline hydration. A markedly enhanced risk reduction for “brief infusion” bicarbonate protocol compared to “long-infusion” one was observed, thereby supporting the findings of Tamura et al. [184]. CM safety committee warrants more studies to be undertaken to assess the effectiveness of single bolus of sodium bicarbonate just before CM administration. If validated, this protocol would be extremely useful in daily clinical practice.

### 12.2. Pharmacological Prophylaxis

Various drugs have been assessed as prophylactic nephroprotective agents against CI-AKI such as N-acetylcysteine (NAC) [71, 186], statins [187, 188], ascorbic acid [189–194], and theophylline [195]. But none of these have so far been approved for the prevention of CI-AKI. At present, the CM safety committee does not support pharmacological prophylaxis but recommends withdrawal of nephrotoxic drugs before CM administration [66].

## 13. N-Acetylcysteine

NAC gives protection against CI-AKI by supplementation of body's antioxidant capacity [196]. In vitro NAC does so powerfully by scavenging hypochlorous acid and also reacting with hydroxyl radicals [197]. In vivo due to its extensive degradation, it is likely that any antioxidant effect it exerts would be indirect, most likely by inducing glutathione synthesis. Studies suggest that NAC prevents glutathione depletion [198, 199] and increases renal glutathione levels [200]; the latter has been reported to result in attenuation of renal injury in ischemia reperfusion models [200, 201] and recently in CI-AKI [202, 203]. Glutathione generally cannot enter the cell; instead it must be synthesized intracellular from glycine, glutamate, and cysteine [204]. Cysteine provides the active HS group required for the glutathione synthesis and hence is the rate limiting factor in this process. NAC after deacylation forms cysteine which enters the renal cells and serves as a precursor for glutathione generation. In addition to its free radical scavenging properties, NAC also has vasodilatory effects [205–207]. By ameliorating CM-induced vasoconstriction, NAC may therefore exert its nephroprotective role [208]. Increase in medullary blood flow with NAC has been reported [209, 210].

The first clinical use of NAC for CI-AKI was reported by Tepel et al. [71]. Eighty-three patients with chronic renal insufficiency were randomly assigned either to receive oral NAC (600 mg twice daily) and 0.45% saline intravenously, before and after administration of the CM, or to receive placebo and saline. NAC receiving patients had lesser incidence of CI-AKI. Since then numerous studies had assessed the role of NAC against CI-AKI. These studies had been performed predominantly in patients undergoing coronary angiography [211]. Some 17 meta-analyses have been published on this subject [211–227], 10 of which favor its use (most of which were published early on). Most of these meta-analyses have reported significant heterogeneity and bias, making it difficult to synthesize clinical treatment recommendation based on the available data. To resolve this heterogeneity, Gonzales et al. performed a meta-analysis of 22 studies using unsupervised clustering, grouping the included trials into two distinct, significantly different (*P* < 0.0001) and homogeneous populations (*P* > 0.5 for both) [224]. Eighteen studies constituted cluster 1, showing no benefit from NAC (*P* = 0.28). Only four studies constituted cluster 2 (making up only 11% of the total meta-analysis), showing significant benefit (*P* = 0.0001). This benefit was observed to be unexpectedly associated with NAC-induced decreases in SCr from baseline. In view of previous reports that NAC in the absence of CM has been shown to decrease SCr levels in normal volunteers [228] and patients [229], this response to NAC may be a drug effect independent of changes in GFR. It was also noted that studies in cluster 2 were relatively early, small, and of lower quality compared with cluster 1 studies (*P* = 0.01 for the three factors combined). Vaitkus and Brar found a significant publication bias throughout the duration in which NAC was being assessed as a nephroprotective agent in CI-AKI, with bias being magnified by meta-analyses [230]. Published manuscripts were observed to present a treatment-effect estimate which was more optimistic than that found in unpublished abstracts. A temporal trend was noted in that the estimate of treatment effect was greatest with early publications, which diminished as additional data became available. Exclusive meta-analyses on oral [220] and intravenous use [227] of NAC also do not support its use as an adjunct to saline hydration. Recently results of the largest multicentre RCT of 2308 patients called “Acetylcysteine for Contrast-induced nephropathy Trial” (ACT) have been published [231]. It randomized patients in 46 centers in Brazil, to receive 1200 mg of oral NAC or placebo twice daily for 2 doses before and after procedure. Intravenous hydration with normal saline, 1 mL/kg/hr, from 6–12 hr before to 6–12 hr after angiography, was strongly recommended. However, changes in the total volume or speed of administration were permitted. The inability of NAC to significantly reduce the incidence of CI-AKI (12.7% in the NAC group and 12.7% in the control group, *P* = 0.97) was evident [186]. Following the above discussion, case for conducting further RCTs using NAC is rather weak.

## 14. Ascorbic Acid

Ascorbic acid acts as an antioxidant [232]. It does so by reacting with most other biologically relevant radicals and oxidants such as hydroxyl ion and superoxide ions to name a few [233]. It readily donates an electron to potentially damaging oxidizing radicals [234]; this one-electron oxidation of AH^−^ results in the production of the ascorbyl radical (A^•−^) also called semidehydroascorbic acid [235]. As a result the reactive free radical is reduced [236]. Ascorbic acid has also been reported to cause vasodilatation in coronary [237] and brachial arteries [238]. Thus vitamin C may have favorable effects on vascular dilatation, possibly through its antioxidant effects on nitric oxide, but these findings are not consistent [239]. Moreover, in most studies, the vitamin C-induced effects on vasodilatation occurred when vitamin C was administered intra-arterially. Through which pathway Vitamin C may afford nephroprotection against CI-AKI remains uninvestigated.

The first clinical use of ascorbic acid for CI-AKI was reported by Spargias et al. [189]. Two hundred and thirty one patients were recruited and randomized to receive either 3 gm of ascorbic acid supplied in chewable tablets or placebo at least 2 hr before the start of the index procedure, followed by 2 gm of ascorbic acid or placebo the night and the morning after the procedure. Intravenous hydration with 50 to 125 mL/hr normal saline was started in all patients from randomization until at least 6 hours after the procedure. Incidence of CI-AKI was lower in ascorbic acid group (9%) and 20% in control group (*P* = 0.02). A significant change in the antioxidant status was observed in the treatment arm. Since then various RCTs have been performed [189, 192, 240–246]. Pooled analysis of these trials has suggested that patients receiving ascorbic acid have 33% less risk of CI-AKI compared to patients receiving placebo or alternate pharmacological treatment (RR: 0.67 (95% CI: 0.46–0.96), *P* = 0.03) [247]. This indicates that ascorbic acid provides effective nephroprotection against CI-AKI and may form a part of effective prophylactic pharmacological regimens. However, use of ascorbic acid has not been recommended by the CM safety committee.

## 15. Statins

Besides their cholesterol lowering properties, statins possess pleiotropic effects that include enhancement of endothelial NO production [248–250] and anti-inflammatory [251] and anti oxidative actions [252, 253]. Statins may also modulate renal hypoperfusion occurring after CM administration by downregulating angiotensin receptors and decreasing the synthesis of endothelin [254]. Due to these properties statins have the potential to be used as nephroprotective agents against CI-AKI. Various trials have assessed this use of statins [255–261]. Pooled analysis of these trials suggests that use of short-term high-dose statin treatment is associated with a significant reduction in risk of CI-AKI (RR = 0.51, 95% CI 0.34–0.76, *P* = 0.001) [262]. There was no evidence of heterogeneity in this meta-analysis. A large multicentre trial is warranted before its routine use as nephroprotective agent can be recommended.

## 16. Theophylline

Adenosine has been implicated to be responsible for mediating CM-induced renal vasoconstriction [263–265] hence use of adenosine antagonists is but logical [266, 267]. Theophylline and aminophylline have been most often used to assess their efficacy as adenosine receptor antagonists in protecting against CI-AKI. Various RCTs have used theophylline [161, 266, 268–281]. A meta-analysis of these trials suggests that theophylline significantly decreases the risk of CI-AKI (RR: 0.48; 95% CI: 0.26–0.89; *P* = 0.02). There was moderate heterogeneity (I^2^ = 45%), suggesting cautious interpretation of these results. Moreover, patients with baseline renal impairment did not show any benefit from theophylline. Multicentre RCTs with large sample size evaluating clinically relevant clinical outcomes are therefore warranted.

## 17. Targeted Renal Therapy

One proposed theory for failure of various drugs used for renal protection is that systemically administered drugs may not be achieving adequate enough drug level in the renal vasculature to be effective against CI-AKI. This has led to the ingenious technique of direct infusion of a drug selectively into the kidneys via the renal arteries, termed targeted renal therapy (TRT). This should have the potential of reducing the systemic side effects of that drug. Fenoldopam, being a dopamine-1 agonist, acts as a vasodilator and hence a potential to attenuate CM-induced cortical and medullar vasoconstriction. Although it was not possible to demonstrate its benefit in reducing prevalence of CI-AKI [282], it was observed that a significant number of patients were not able to tolerate low doses of fenoldopam due to drug induced hypotension, which is itself a risk factor of CI-AKI. Employing TRT, selective bilateral renal artery catheterization may be performed for localized drug delivery. In a pilot feasibility study on patients undergoing endovascular aneurysm repair, Benephit PV Infusion System (Flowmedica, Inc., Fremont, CA, USA) was used for selective catheterization of bilateral renal arteries via brachial artery puncture. There were no catheter migrations, thrombosis, device-related complications, or vascular access complications. There was no episode of hypotension, so all patients received fenoldopam at a rate of 0.4 *μ*g/kg/min for the duration of the aneurysm repair [283]. If the pigtail catheter is just kept in aorta just above the level of renal arteries rather than selective catheterization of renal artery, this may seem a simpler approach but would lead to significant systemic drug effects due to direct delivery of the drug into systemic circulation [283]. The safety and performance of TRT were also assessed by retrospective analysis of 285 patients receiving fenoldopam via TRT, as a part of “The Benephit System Renal Infusion Therapy (Be-RITe)” registry [284]. Benephit Infusion System (Flowmedica, Inc., Fremont, CA, USA) was used. Bilateral renal artery cannulation was successful in 94.2%, with a mean cannulation time of 2.0 min. Incidence of CI-AKI was 71% lower than predicted, with significant benefit in patients with highest risk of CI-AKI. Prospective studies and RCTs are therefore warranted to assess true potential of this technique. The TRT delivery system lacks cost effectiveness [hospital cost of the Benephit System is ~$1,100.00; the periprocedural fenoldopam cost is ~$100.00]. In the tough time of economic meltdown and spending cuts in healthcare systems, strong evidence would be required before it can be considered for use in clinical practice.

## 18. Ischemic Preconditioning

Ischemic preconditioning involves exposure to brief episodes of ischemiareperfusion to prepare target organ against the main ischemic insult. If the site of generation of these brief episodes of ischemic reperfusion is distant from the site of target organ, it is called remote ischemic preconditioning. This technique has been used with only variable success in affording myocardial and renal protection in cardiovascular medicine and surgery [90, 285–291]. Recently, results of an RCT suggest benefit from remote ischemic preconditioning in preventing CI-AKI [292]. The likely benefit may stem from its ability to attenuate the CM-induced ischemia reperfusion injury. However in the recent years ischemic preconditioning has lost its glamour due to the failure to translate successful results in laboratory settings into clinical practice [293]. With this in mind, large multicentre trials with clinically relevant outcomes of renal function are warranted to assess its role in CI-AKI prophylaxis.

## 19. Conclusions

CI-AKI remains a widely debated topic today with its pathophysiology still under investigation. There is strong evidence though about the use of LOCM and IOCM rather than HOCM due to their improved safety profile. However, CM dose should be kept to the minimum as no CM is 100% safe and can lead to acute kidney injury. SCr continues to be the widely used laboratory test for defining CI-AKI whereas eGFR is used for grading the severity of renal impairment. Although novel urinary and serum biomarker levels vary with acute kidney injury, their modulation has failed to consistently yield clinically relevant outcomes such as reduction in incidence of CI-AKI or need for dialysis. Future research into the development of such biomarkers of renal injury should seriously consider these limitations. Among the prophylactic strategies against CI-AKI, volume expansion using oral hydration and/or intravenous normal saline hydration with or without sodium bicarbonate supplementation remains the gold standard. Strict avoidance of nephrotoxic drugs such as NSAIDs must be adhered to before CM exposure. The evidence for use of pharmacological agents against CI-AKI such as NAC, ascorbic acid, theophylline/aminophylline, statins, targeted renal therapy, and ischemic preconditioning is not robust; hence no recommendations exist for their routine clinical use. Rather than performing a small sample size trial at a single institution, large multicentre adequately powered RCTs should be organized by collaborative efforts among interested investigators, to demonstrate clinically relevant outcomes in order to successfully combat the long-standing menace of CI-AKI.
